# Effect of Positive End-Expiratory Pressure on Central Venous Pressure in Patients under Mechanical Ventilation

**Published:** 2017-01-08

**Authors:** Majid Shojaee, Anita Sabzghabaei, Hossein Alimohammadi, Hojjat Derakhshanfar, Afshin Amini, Bahareh Esmailzadeh

**Affiliations:** 1 Department of Emergency Medicine, Imam Hossein Hospital, Shahid Beheshti University of Medical Sciences, Tehran, Iran.; 2 Department of Emergency Medicine, Loghmane Hakim Hospital, Shahid Beheshti University of Medical Sciences, Tehran, Iran.

**Keywords:** Positive-pressure respiration, central venous pressure, ventilators, mechanical, catheterization, central venous, emergency service, hospital

## Abstract

**Introduction:**

Finding the probable governing pattern of PEEP and CVP changes is an area of interest for in-charge physicians and researchers. Therefore, the present study was designed with the aim of evaluating the relationship between the mentioned pressures.

**Methods:**

In this quasi-experimental study, patients under mechanical ventilation were evaluated with the aim of assessing the effect of PEEP change on CVP. Non-trauma patients, over 18 years of age, who were under mechanical ventilation and had stable hemodynamics, with inserted CV line were entered. After gathering demographic data, patients underwent 0, 5, and 10 cmH_2_O PEEPs and the respective CVPs of the mentioned points were recorded. The relationship of CVP and PEEP in different cut points were measured using SPSS 21.0 statistical software.

**Results:**

60 patients with the mean age of 73.95 ± 11.58 years were evaluated (68.3% male). The most frequent cause of ICU admission was sepsis with 45.0%. 5 cmH_2_O increase in PEEP led to 2.47 ± 1.53 mean difference in CVP level. If the PEEP baseline is 0 at the time of 5 cmH_2_O increase, it leads to a higher raise in CVP compared to when the baseline is 5 cmH_2_O (2.47 ± 1.53 vs. 1.57 ± 1.07; p = 0.039). The relationship between CVP and 5 cmH_2_O (p = 0.279), and 10 cmH_2_O (p = 0.292) PEEP changes were not dependent on the baseline level of CVP.

**Conclusion:**

The findings of this study revealed the direct relationship between PEEP and CVP. Approximately, a 5 cmH_2_O increase in PEEP will be associated with about 2.5 cmH_2_O raise in CVP. When applying a 5 cmH_2_O PEEP increase, if the baseline PEEP is 0, it leads to a significantly higher raise in CVP compared to when it is 5 cmH_2_O (2.5 vs. 1.6). It seems that sex, history of cardiac failure, baseline CVP level, and hypertension do not have a significant effect in this regard.

## Introduction:

A vast number of patients visiting emergency department (ED) need intubation due to their level of consciousness and clinical status, and are kept under mechanical ventilation. These patients may also require central venous (CV) line insertion for different reasons such as inability to access peripheral vein, blood transfusion, or administration of vasoactive agents (1). Central venous pressure (CVP) is a good indicator of circulatory volume and cardiac function, which may be influenced by various factors such as function of right atrium and ventricle, venous tone, and intra-thoracic pressure (1). Considering the afore-mentioned utilities, CVP monitoring is routinely used in operating rooms, intensive care units (ICU), and EDs. The normal range of CVP is 8 - 12 cmH_2_O, which increases to 12 - 16 cmH_2_O in patients under mechanical ventilation (1, 2). Factors such as incorrect adjustment of the ruler’s zero point, patient’s poor condition, inappropriate placement of the catheter, and using vasopressor may interfere with accurate CVP measurement (1). Positive end-expiratory pressure (PEEP) in patients under mechanical ventilation can affect CVP via increasing intra-thoracic pressure. Various reports exist on the direct relationship between the 2 pressures (3). Yet, there is no accurate formula or solution based on PEEP level for adjusting CVP in patients under mechanical ventilation. Yang et al. showed that a 0.38 cmH_2_O increase in PEEP leads to 1 cmH_2_O raise in CVP (4). A study on 70 cardiac surgery patients in 2007 showed that mean CVP of the patients in 0, 5, and 10 cmH_2_O PEEPs are 11, 12, and 14 cmH_2_O, respectively (5).

Finding the probable governing pattern of PEEP and CVP changes is an area of interest for in-charge physicians and researchers. Therefore, the present study was designed with the aim of evaluating the relationship between the mentioned pressures.

## Methods:

***Study design and setting***

In this quasi-experimental study, patients under mechanical ventilation who were hospitalized in the ICU of Imam Hossein Hospital, Tehran, Iran, were evaluated with the aim of assessing the effect of PEEP change on CVP. The study was approved by the Ethics Committee of Shahid Beheshti University of Medical Sciences in their 147^th^ meeting in February 2013. Since the patients were not able to give written informed consent, it was obtained from their relatives. The researchers were committed to protecting patient rights and confidentiality in line with the principles of Helsinki Declaration. 

***Participants***

Non-trauma patients, over 18 years of age, who were under mechanical ventilation and had stable hemodynamics, with inserted CV line were entered. To eliminate other confounding factors of CVP, all the patients were deeply sedated and under the same ventilator settings including tidal volume = 8 – 10 cc/kg, pressure support = 12 – 14 cmH_2_O, FiO_2_ = 40-70%, flow = 10 – 12 l/minute, and PEEP = 0, 5, and 10 cmH_2_O. Intake volume per hour was in a similar range for all the patients and the patient was excluded if more than 200 cc/hour intravenous isotonic fluid was needed to maintain hemodynamic stability. Patients with auto PEEP (more than 2 cmH_2_O difference between the PEEP reported by the device and the one set for the patient), whatever the reason, were excluded. In addition, patients who were in need of ˃ 10 cmH_2_O PEEP, for example patients with acute respiratory distress syndrome (ARDS), were not evaluated. The ventilator devices used for the patients were all the same model and from the same manufacturer.

***Data gathering***

After gathering demographic data of the patients using a checklist designed for this study, patients underwent 0, 5, and 10 cmH_2_O PEEPs and the respective CVPs of the mentioned points were measured and recorded. The time considered for adjustment of CVP with any of the PEEP cut-off points was considered 10 minutes (3). To accurately measure CVP, all measurements were carried out by the same person, in supine position, and by setting the zero point of the CV line ruler at sternal notch level. To minimize errors, measurement for every patient was done twice for each PEEP cut point with 30 minutes intervals and their mean was considered the reference CVP measure. In cases of wide difference between the 2 measurements, a third measurement was done and the mean of the 2 closest measures was considered to be the reference. If a participant showed hypoxia and hemodynamic instability at any time of the study, he/she was eliminated from the study and necessary interventions were carried out to stabilize his/her status. All the calculated measures for CVP in each PEEP cut point was recorded in the prepared checklist and used for analysis.

***Statistical analyses***

The sample size needed for the present study was calculated based on a pilot study and using a standard deviation to estimate the minimum sample size needed. Therefore, by considering Zα = 0.5%, p = 95%, minimum clinically considerable CVP difference of 1 cmH_2_O (d = 1), and the difference between the standard deviation of CVP in PEEP 10 and 0 of 1.4 (*S*_diff _= 1.4), the sample size needed was calculated to be 26 cases. Patient data were analyzed using SPSS 21.0. Quantitative data were reported as mean and standard deviation and qualitative ones as frequency and percentage. To compare CVP before and after applying various PEEPs, paired t-test or non-parametric Wilcoxon test were used. In all tests, p < 0.05 was considered as significance level. 

## Results

60 patients with the mean age of 73.95 ± 11.58 years (46 – 93) were evaluated (68.3% male). The most frequent cause of ICU admission was sepsis with 45.0%. Table 1 shows the baseline characteristics of the studied patients. Table 2 shows the relationship between various PEEP measures and CVP. 5 cmH_2_O increase in PEEP led to 2.47 ± 1.53 mean difference in CVP level. If the PEEP baseline is 0 at the time of 5 cmH_2_O increase, it leads to a higher raise in CVP compared to when the baseline is 5 cmH_2_O (2.47 ± 1.53 vs. 1.57 ± 1.07; p = 0.039). Adjusting the analyses done in table 2 based on sex, presence of cardiac failure and history of hypertension did not show any significant differences in the mentioned relations (table 3). Evaluation of the relationship between changes in PEEP and CVP measures based on different levels of CVP are summarized in table 4.

The relationship between CVP and 5 cmH_2_O (p = 0.279), and 10 cmH_2_O (p = 0.292) PEEP changes were not dependent on the baseline level of CVP.

**Table1:** Baseline characteristics of the studied population

**Studied variables**	**Mean ± Standard deviation (range)**
**Systolic blood pressure (mmHg)**	125.533 ± 20.55 (100 – 170)
**Diastolic blood pressure (mmHg)**	74.583 ± 11.21 (60 – 100)
**Heart rate (beats/minute)**	90.96 ± 15.34 (60 – 120)
**Age (years)**	**Frequency (%)**
18 – 39.9	0 (0)
40 – 59.9	7 (11.7)
60 – 79.9	29 (48.3)
≥ 80	24 (40)
**History of cardiac failure**	
Yes	12 (20)
No	48 (80)
**History of hypertension **	
Yes	43 (71.7)
No	17 (28.3)
**Cause of ICU admission**	
Pneumosepsis	14 (23.3)
Urosepsis	13 (21.7)
CVA	8 (13.3)
Metastatic cancer	4 (6.7)
Other	21 (35)

ICU: intensive care unit; CVA: cardiovascular accident.

**Table 2:** Correlation between different positive end-expiratory pressures (PEEP) and central venous pressures (CVP) in studied patients

**PEEP (cmH**_2_ **O) **	**CVP (mean ± SD) (cmH**_2_ **O)**	**P value**
**Paired 1**		
0	7.81 ± 5.99	< 0.001
5	10.29 ± 5.67	
**Paired 2**		
0	7.81 ± 5.99	< 0.001
10	11.86 ± 5.72	
**Paired 3**		
∆ 0-5	2.47 ± 1.53	< 0.001
∆ 0-10	4.05 ± 2.09	
**Paired 4**		
∆ 0-5	2.47 ± 1.53	0.039
∆ 5-10	1.57 ± 1.07	

SD: standard deviation.

**Table 3:** Correlation between different positive end-expiratory pressures (PEEP) and sex, and history of heart failure and hypertension

	**Sex** *		**Heart failure** **		**Hypertension** ***	
**PEEP ** **(cmH**_2_ **O)**	**Mean ± SD**	**P value**	**Mean ± SD**	**P value**	**Mean ± SD**	**P value**
**PEEP 0**						
0	7.23 ± 5.00	0.27	7.22 ± 5.79	0.13	8.02 ± 1.14	0.86
1	9.07 ± 7.73		10.16 ± 6.46		7.73 ± 4.81	
**PEEP 5**						
0	9.74 ± 4.73	0.27	9.94 ± 5.48	0.35	10.61 ± 7.92	0.78
1	11.47 ± 7.32		11.66 ± 6.47		10.16 ± 4.61	
**PEEP 10**						
0	11.54 ± 4.97	0.53	11.60 ± 5.52	0.48	11.91 ± 7.72	0.97
1	12.55 ± 7.20		12.91 ± 6.64		11.84 ± 4.83	
**∆ 0-5**						
0	2.51 ± 1.66	0.78	2.71 ± 1.55	0.01	2.58 ± 1.20	0.72
1	2.39 ± 1.23		1.50 ± 0.97		2.43 ± 1.65	
**∆ 5-10**						
0	1.80 ± 1.15	0.01	1.65 ± 1.06	0.24	1.29 ± 0.83	0.20
1	1.07 ± 0.65		1.25 ± 1.07		1.68 ± 1.14	
**∆ 0-10 **						
0	4.31± 2.24	0.14	4.37 ± 2.12	0.01	3.88 ± 1.63	0.70
1	3.47 ± 1.61		2.75 ± 1.37		4.11 ± 2.26	

* Sex (0 = Male, 1 = Female),

** Heart failure (0 = No, 1 = Yes),

*** Hypertension (0 = No, 1 = Yes).

**Table 4:** Comparison of correlation between positive end-expiratory pressures (PEEP) and central venous pressure (CVP) in different baseline CVP levels

**PEEP (cmH**_2_ **O)**	**∆ CVP (cmH**_2_ **O) **	**P value**
**∆ PEEP 5 **		
CVP < 8	2.7 ± 1.7	0.279
CVP = 8 -12	2.4 ± 1.2	
CVP > 12	1.8 ± 1.2	
**∆ PEEP 10**		
CVP < 8	4.3 ± 2.3	0.292
CVP = 8 -12	4.1 ± 1.3	
CVP > 12	3.1 ± 1.9	

## Discussion

The findings of this study showed that an increase in PEEP has a direct relationship with CVP increase. Approximately, a 5 cmH_2_O increase in PEEP will be associated with about 2.5 cmH_2_O raise in CVP. When applying a 5 cmH_2_O PEEP increase, if the baseline PEEP is 0, it leads to a significantly higher raise in CVP compared to when it is 5 cmH_2_O (2.5 vs. 1.6). It seems that sex, history of cardiac failure, hypertension, and baseline CVP do not significantly affect CVP increase rate. 

In a study by Yang et al. 1 cmH_2_O increase in PEEP, led to 0.38 cmH_2_O increase in CVP, which is approximately in line with the present study (4). A study on the effect of PEEP in patients under mechanical ventilation showed a significant direct relationship between 0, 5, and 10 cmH_2_O PEEPs with CVP. The CVP increase was related to mean PEEP during mechanical ventilation when PEEP was set 10 or less, in a study by Cao et al., which is in line with this study (6). Evaluating the effect of PEEP on CVP and stroke volume in 20 patients with cardiac diseases, revealed that PEEP significantly affects CVP, while no significant relationship was detected between heart rate and mean arterial pressure (7). In the present study, 5 cmH_2_O increase in PEEP, led to 1.5 – 2.5 cmH_2_O increase in CVP. Currently, CVP is used as a guide for fluid therapy efficiency and monitoring the effects of intake volume on cardiovascular system. Many current treatment protocols, especially regarding septic shock patient management, define the aim of the treatment as achieving a CVP of 8 - 12 cmH_2_O in patients without ventilator and 12 - 16 cmH_2_O in those under mechanical ventilation. However, the study by Cao et al. showed that in patients under ventilator and PEEP, CVP alone is not a good reference for estimation of circulatory volume and required fluid volume for resuscitation (8). Considering the existing controversies in this regard, it seems that we should seek more accurate scales for determining the efficacy of fluid therapy in patients under mechanical ventilation. Until then, the best way might be using modified CVP based on PEEP rate.

## Limitations:

Since the present study was carried out on patients hospitalized in the ICU, some limitations should be noted: First, patients with a variety of underlying illnesses were included, which can affect the results. Second, the patients have been in different phases of hospitalization, therefore the rates and efficiencies of treatments received (fluid, vasoactive drugs) were different among them. In addition, CVP measurement using a ruler has some limitations in its nature, such as adjusting the zero point. Naturally, there are some limitations for applying long-term PEEPs in these patients, which can affect the conclusion. 

It is suggested to eliminate the afore-mentioned limitations to accurately evaluate the effect of PEEP on CVP in future studies.

## Conclusion:

The findings of this study revealed the direct relationship between PEEP and CVP. Approximately, a 5 cmH_2_O increase in PEEP will be associated with about 2.5 cmH_2_O raise in CVP. When applying a 5 cmH_2_O PEEP increase, if the baseline PEEP is 0, it leads to a significantly higher raise in CVP compared to when it is 5 cmH_2_O (2.5 vs. 1.6). It seems that sex, history of cardiac failure, baseline CVP level, and hypertension do not have a significant effect in this regard.
